# Regional Intraosseous Administration of Prophylactic Antibiotics is More Effective Than Systemic Administration in a Mouse Model of TKA

**DOI:** 10.1007/s11999-015-4464-x

**Published:** 2015-07-30

**Authors:** Simon W. Young, Tim Roberts, Sarah Johnson, James P. Dalton, Brendan Coleman, Siouxsie Wiles

**Affiliations:** Department of Surgery, University of Auckland, Auckland, New Zealand; Department of Orthopaedic Surgery, North Shore Hospital, 124 Shakespeare Road, Takapuna, Private Bag 93-503, Auckland, 0740 New Zealand; Bioluminescent Superbugs Laboratory, Faculty of Medical & Health Sciences, University of Auckland, Auckland, New Zealand; Maurice Wilkins Centre for Molecular Biodiscovery, Auckland, New Zealand

## Abstract

**Background:**

In human TKA studies, intraosseous regional administration (IORA) of prophylactic antibiotics achieves local tissue antibiotic concentrations 10 times greater than systemic administration. However, it is unclear if such high concentrations provide more effective prophylaxis.

**Questions/purposes:**

We asked: (1) What prophylaxis dosage and route (intravenous [IV] versus IORA of prophylactic antibiotics) produce less in vivo bacterial burden compared with no-antibiotic controls? (2) Compared with controls, what prophylaxis dosage and route yield fewer colony-forming units (CFUs) in euthanized animals in a model of TKA? (3) Is prophylactic IORA of antibiotics more effective than same-dose IV antibiotic administration in reducing CFUs?

**Methods:**

Mice (six to nine per group) were block randomized to one of six prophylaxis regimens: control, systemic cefazolin (C100_IV_), IORA of cefazolin (C100_IORA_), systemic vancomycin (V110_IV_), low-dose systemic vancomycin (V25_IV_), and low-dose IORA of vancomycin (V25_IORA_). Surgery involved placement of an intraarticular knee prosthesis, followed by an inoculum of bioluminescent *Staphylococcus aureus* strain Xen36. Biophotonic imaging assessed in vivo bacterial loads, and after 4 days bacterial load was quantified using culture-based techniques. Comparisons were made for each prophylactic regimen to controls and between same-dose IV and IORA of prophylactic antibiotic regimens.

**Results:**

Mice treated with systemic high-dose vancomycin, IORA of vancomycin, or IORA of cefazolin had lower in vivo *Staphylococcus aureus* burdens (median area under curve, Control: 5.0 × 10^6^; V110_IV_: 1.5 × 10^6^, difference of medians 3.5 × 10^6^, p = 0.003; V25_IV_: 1.94 × 10^6^, difference 3.07 × 10^6^, p = 0.49; V25_IORA_: 1.51 × 10^6^, difference 3.5 × 10^6^, p = 0.0011; C100_IORA_: 1.55 × 10^6^, difference 3.46 × 10^6^, p = 0.0016; C100_IV_: 2.35 × 10^6^, difference 2.66 × 10^6^, p = 0.23.) Similar findings were seen with culture-based techniques on recovered implants. IORA of prophylactic antibiotics was more effective than same-dose IV administration in reducing bacterial load on recovered implants (median CFUs < 7.0 × 10^0^ vs 2.83 × 10^2^, p = 0.0183).

**Conclusions:**

IORA of prophylactic cefazolin and vancomycin was more effective than the same dose of antibiotic given systemically. The effectiveness of vancomycin in particular was enhanced by IORA of prophylactic antibiotics despite using a lower dose.

**Clinical relevance:**

Our study supports previous studies of IORA of prophylactic antibiotics in humans and suggests this novel form of administration has the potential to enhance the effectiveness of prophylaxis in TKA. Because of concerns regarding antibiotic stewardship, IORA of prophylactic vancomycin may be more appropriately restricted to patients having TKA who are at greater risk of infection, and clinical trials are in progress.

## Introduction

Prophylactic antibiotics aim to provide protection against the bacteria most likely to cause contamination during surgery [5, 57]. The two most common bacteria causing contamination and subsequent deep infection in TKAs are *Staphylococcus aureus* and coagulase-negative staphylococci [14, 26, 37]. In the 1960s and 1970s when preoperative prophylactic antibiotics were introduced, as much as 98% of hospital isolates of coagulase-negative staphylococci and 97% of *S aureus* were sensitive to cephalosporins [18, 27, 33, 42], and cephalosporins subsequently became the commonly recommended agent for prophylaxis in arthroplasty [7, 11, 23, 25]. Currently however, as much as 90% of hospital coagulase-negative staphylococci isolates are resistant to cephalosporins [14, 26, 37, 50, 55], and 30% to 56% of *S aureus* cultured from infected joint arthroplasties are methicillin-resistant (MRSA) [30, 31, 34, 39]. Vancomycin has been suggested as an alternative prophylactic agent, as currently it remains effective against MRSA and coagulase-negative staphylococci resistant to cefazolin [4, 49]. However injudicious use of vancomycin may risk further resistance, and in clinical studies it is a less-effective prophylactic agent than cefazolin against methicillin-sensitive *S aureus* strains (MSSA) [4, 19]. This may be because adequate vancomycin tissue levels are not achieved with typical systemic doses [22, 42], particularly when timing of prophylactic administration is suboptimal [4, 17].

Higher tissue levels of antibiotic can be achieved with alternative methods of administration. Intraosseous regional administration (IORA) of prophylactic antibiotics is a novel form of administration that involves intraosseous injection after tourniquet inflation but before skin incision. In a randomized trial of patients who had TKAs comparing 1g cefazolin given by IORA or systemic routes, IORA achieved 10 times greater antibiotic tissue concentrations [57]. IORA also achieves high tissue concentrations when lower doses of prophylactic antibiotic are used [56], an advantage for agents such as vancomycin where systemic toxicity including red man syndrome is a concern [9]. However it is unclear if these high tissue concentrations seen in clinical studies of IORA using either vancomycin or cefazolin provide more effective prophylaxis against infection.

The aim of our study was to compare the effectiveness of prophylactic IORA of antibiotics with systemic administration using an in vivo murine model of TKA [38]. Specifically, we asked: (1) What antibiotic administration dosage and route (intravenous [IV] versus IORA) produce less in vivo bacterial burden compared with no-antibiotic controls? (2) Compared with controls, what prophylactic antibiotic administration dosage and route yield fewer colony-forming units (CFUs) in euthanized animals in a model of TKA? (3) Is prophylactic IORA more effective than same-dose IV antibiotic administration in reducing CFUs?

## Materials and Methods

### Bioluminescent *S aureus*

Bioluminescent MSSA Xen36 [6] (Perkin Elmer, Waltham, MA, USA) was used in all experiments. Xen36 is a derivative of clinical bacteremia isolate ATCC 49525 (Wright) with a modified *lux* operon from *Photorhabdus luminescens* stably integrated in a native plasmid [6].

Bacteria were grown overnight in Tryptic soy broth (Fort Richard Laboratories Ltd, Auckland, New Zealand) at 37 °C with shaking at 200 rpm, then reinoculated in fresh media at 1:5 and incubated for an additional 90 minutes. Bacteria then were checked for light expression, washed three times in phosphate-buffered saline (PBS), and resuspended in PBS to obtain approximately 5 × 10^9^ CFU/mL. The concentration of bacteria in solution was verified retrospectively by plating and culture.

### Animals

**Fig. 1:**
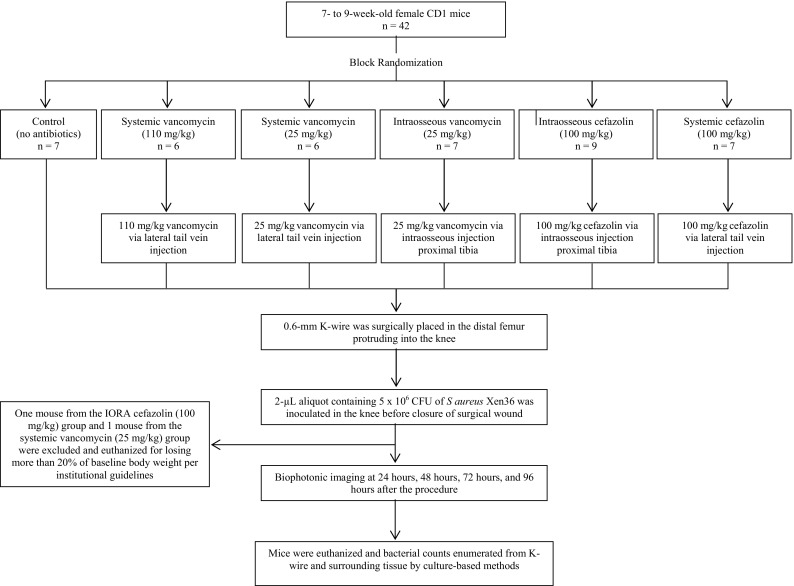
A schematic of the experimental design we used in this study is shown.

Female CD1 mice were obtained from the specific pathogen-free breeding facility at the University of Auckland. The mice were 7 to 9 weeks old on arrival and were given food and water ad libitum. Animals were housed and cared for in accordance with the New Zealand Animal Welfare Act [36] and institutional guidelines provided by the University of Auckland Animal Ethics Committee, which reviewed and approved these experiments under application R1134. As single housing of animals is discouraged, all experiments were performed using female mice, as they are less aggressive than males, and so less likely to injure themselves or each other when housed together. Conditions and diet were identical for all animals. To minimize the number of animals required, while accounting for any host, bacterial, or surgical variation, one experiment was performed using a block design (Fig. 1). Surgery was performed on six separate occasions using a different cohort of mice and a fresh preparation of bacteria. At each surgery six to eight animals were randomized to one of the six experimental groups, to give group sizes of six to eight animals.

### Antibiotic Prophylaxis

Mice were randomized into six experimental groups: (1) no antibiotic prophylaxis (n = 7); (2) systemic vancomycin (110 mg/kg, V110_IV_, n = 6); (3) systemic vancomycin (25 mg/kg, V25_IV_, n = 6); (4) intraosseous vancomycin (25 mg/kg, V25_IORA_, n = 7); (5) intraosseous cefazolin (100 mg/kg, C100_IORA_, n = 9); and (6) systemic cefazolin (100 mg/kg, C100_IV_, n = 7). These experimental groups represent vancomycin IV at either a high therapeutic dose (110 mg/kg) or a suboptimal dose (25 mg/kg) or intraosseously at a low dose (25 mg/kg). We administered cefazolin at a standard therapeutic dose either IV or intraosseously. These reflect the dosages and routes of administration used in two previous human studies of IORA [56, 57]. One mouse from the IORA cefazolin (100 mg/kg) group and one from the systemic vancomycin (25 mg/kg) group were euthanized for losing more than 20% of baseline body weight per institutional guidelines and were excluded from the analysis (Fig. 1).

Antibiotics, when used, were administered either systemically via an IV route or regionally (below a tourniquet) via an intraosseous route. Systemic antibiotics were introduced by injection into the lateral tail vein 30 minutes before surgery. Regional intraosseous antibiotics, however, were administered by direct injection into the proximal tibia after tourniquet inflation to the extremity and immediately before surgery. Antibiotics were given by intraosseous injection into the tibia using a 26-gauge needle as previously described [24, 29, 47]. The 110 mg/kg dose of vancomycin is an effective dose in mice, approximating the area under the curve (AUC) of 400 mg.hour/L for a typical human dose of vancomycin (1 g every 12 hours) [20, 38]. Using the body surface normalization method [44], this represents a human dose of approximately 10 to 15 mg/kg. We used an IORA dose of vancomycin that was approximately 25% of this, as reported in a human study, where a lower dose was used to protect against systemic effects such as red man syndrome [56]. Because cefazolin has minimal systemic toxicity, we used the same dose for the systemic and IORA routes as in a previous IORA study of humans [57]. The cefazolin dose of 100 mg/kg in mice gives serum concentrations similar to a 1- to 2-g prophylactic dose in humans [8, 28, 54].

### Surgical Procedure

Mice were weighed preoperatively and inhalational isoflurane (3.0%) was administered for anesthesia. In the absence of a toe pinch reflex, the right leg was depilated using clippers and an above-knee tourniquet was applied. The surgical site was prepared using an iodine-povidone swab followed by an alcohol swab and a final iodine-povidone wash.

The knee was accessed using a medial parapatellar approach and the intercondylar region of the distal femur identified. The femoral medullary canal was reamed manually with sequentially larger-gauge needles for the stainless steel implant, starting with a 26-gauge needle. A sterile 0.6-mm K-wire then was inserted in a retrograde fashion through the intercondylar region into the intramedullary cavity of the distal femur. The K-wire was cut with approximately 1 mm of wire protruding in the joint cavity. Before closing, a 2-µL aliquot containing approximately 5 × 10^6^ CFU of *S aureus* Xen36 was pipetted into the joint. The patella complex then was reduced and the incision closed with 6–0 Monocryl^TM^ sutures (Ethicon, Somerville, NJ, USA). The total tourniquet time for each mouse was 30 minutes. Postoperatively, the mice received acetaminophen (paracetamol) (6 mg/mL) in the drinking water and carprofen (5 mg/kg) subcutaneously once daily.

### Biophotonic Imaging

**Fig. 2:**
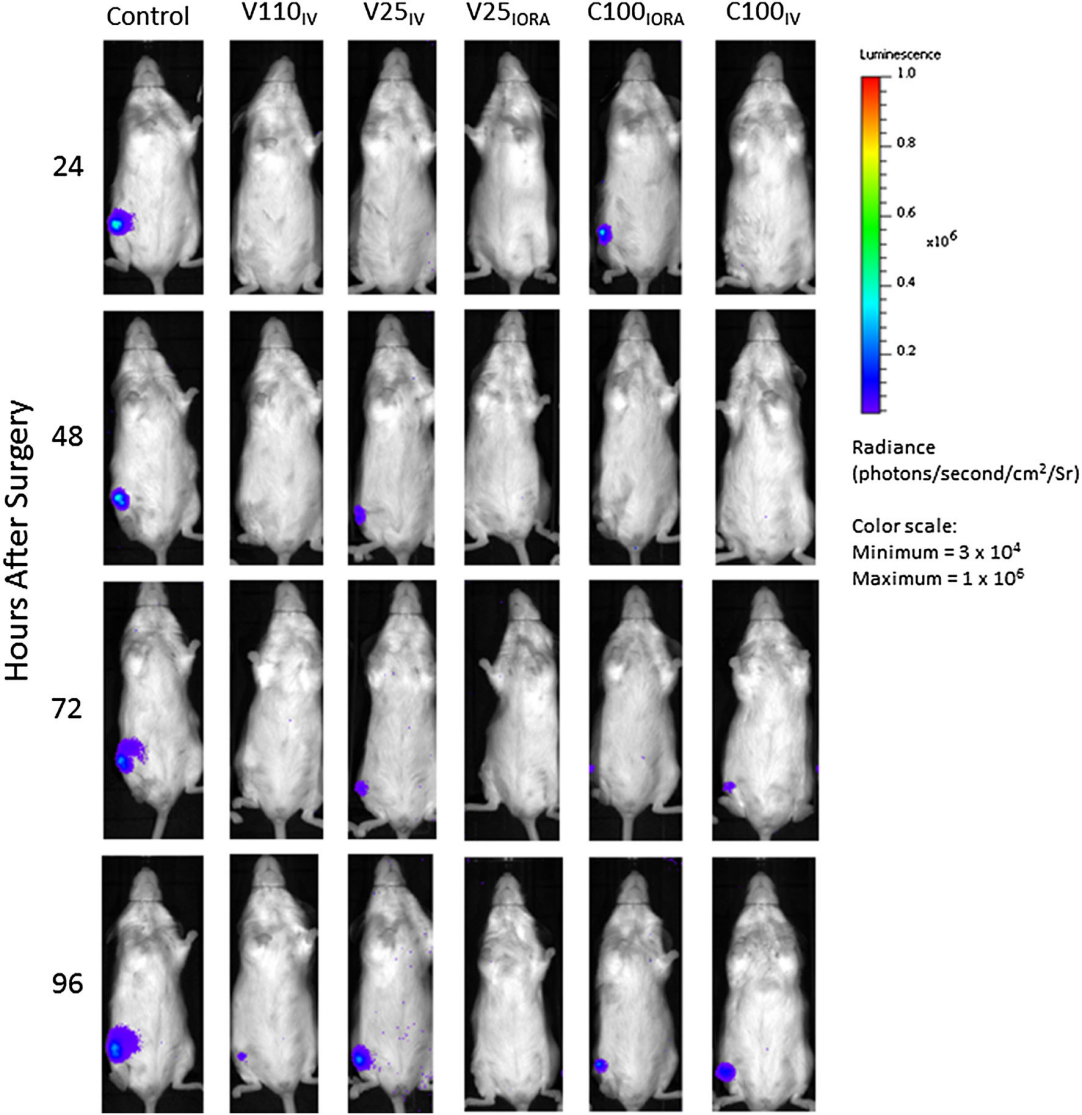
Bioluminescence from *S aureus* Xen36 from anesthetized animals was assessed after surgery. The images show peak bioluminescence with variations in color representing light intensity at a given location. Red represents the most intense light emission, whereas blue corresponds to the weakest signal. The color bar indicates relative signal intensity (as photons/second/cm^2^/steradian [Sr]). Mice were imaged at various times after surgery with an integration time of 5 minutes. One representative animal is shown for each group. IV = intravenous; IORA = intraosseous regional administration; V110_IV_ = systemic vancomycin, 110 mg/kg; V25_IV_ = systemic vancomycin, 25 mg/kg; V25_IORA_ = IORA vancomycin, 25 mg/kg; C100_IORA_ = IORA cefazolin, 100 mg/kg; C100_IV_ = systemic cefazolin, 100 mg/kg.

**Fig. 3A–B:**
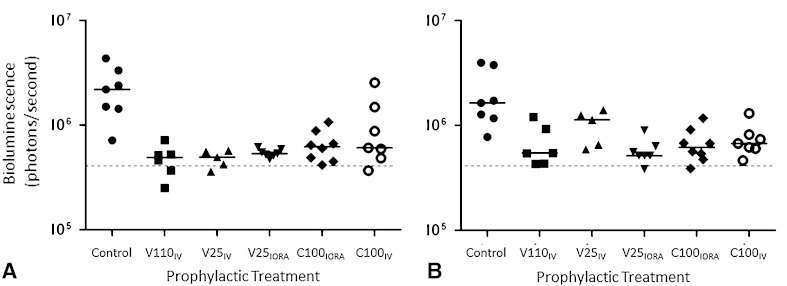
Quantification of bioluminescence from *S aureus* Xen36 from anesthetized animals after surgery is shown. The bioluminescent signals originating from individual animals at (**A**) 1 day and (**B**) 4 days after surgery were obtained using the region of interest tool in the Living Image software program (given as photons/second). The dotted line represents the level of background from uninfected animals. Median values per group are denoted by solid lines. Each symbol represents an individual animal. Data are pooled from six independent repeats with one to two animals per group per repeat. IV = intravenous; IORA = intraosseous regional administration; V110_IV_ = systemic vancomycin, 110 mg/kg; V25_IV_ = systemic vancomycin, 25 mg/kg; V25_IORA_ = IORA vancomycin, 25 mg/kg; C100_IORA_ = IORA cefazolin, 100 mg/kg; C100_IV_ = systemic cefazolin, 100 mg/kg.

**Fig. 4:**
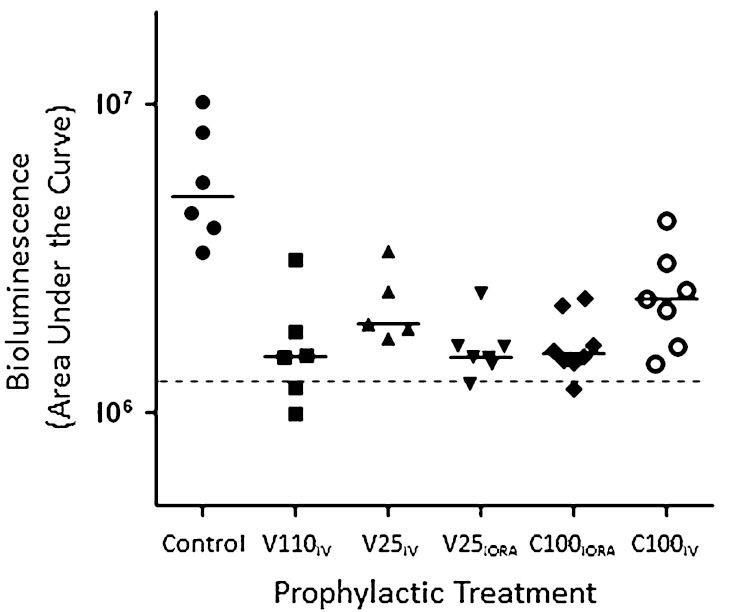
Area under curve values (summation during entire test period) from bioluminescent signals obtained throughout the experiment are shown. The dotted line represents the level of background from uninfected animals. Median values per group are denoted by solid lines. Each symbol represents an individual animal. Data are pooled from six independent repeats with one to two animals per group per repeat. IV = intravenous; IORA = intraosseous regional administration; V110_IV_ = systemic vancomycin, 110 mg/kg; V25_IV_ = systemic vancomycin, 25 mg/kg; V25_IORA_ = IORA vancomycin, 25 mg/kg; C100_IORA_ = IORA cefazolin, 100 mg/kg; C100_IV_ = systemic cefazolin, 100 mg/kg.

Biophotonic imaging was used to noninvasively measure the bioluminescent signal emitted by *S aureus* Xen36 from anesthetized mice to provide information regarding the localization of the bacterium (given as photons per second per square centimeter per steradian [photons second/cm^2^/sr]) (Fig. 2). We also quantified the bacterial burden in vivo from the biophotonic signal of selected regions of interest (given as photons/second) using Living Image software (Perkin Elmer) (Fig. 3). Measurements were obtained daily to present as values for the AUC for each animal (Fig. 4).

Assessment of bioluminescence (photons/second/cm^2^/sr) from living animals was measured after gaseous anesthesia with isoflurane using the IVIS^®^ Kinetic camera system (Perkin Elmer). A photograph (reference image) was taken under low illumination before quantification of photons emitted from Xen36 at a binning of four over 5 minutes using the Living Image software. For anatomic localization, a pseudocolor image representing light intensity (blue, least intense to red, most intense) was generated using the Living Image software and superimposed over the gray-scale reference image. Bioluminescence in specific regions of individual mice also was quantified using the region of interest tool in the Living Image software program (given as photons per second).

### Quantification of Bacteria in the Knee and Implant

Mice were euthanized by cervical dislocation under anesthesia. The hindlimb was surface-sterilized with 70% ethanol and the skin removed. The knee (including approximately 5 mm of the proximal end of tibia and distal end of femur) and surrounding tissue were excised. The K-wire was extracted from the femur and placed in a 1.5-mL microtube containing 0.5 mL PBS. The excised knee was placed in a 2-mL sample tube containing ceramic beads and 1 mL PBS and homogenized (3 × 10 seconds at 3.55 m/second) using a tissue disruptor (OMNI International, Kennesaw, GA, USA). Serial dilutions were plated on Mannitol salt agar (Fort Richard Laboratories Ltd) and grown overnight at 37 °C for viable count enumeration. Plates subsequently were imaged with the IVIS^®^ Kinetic camera system to confirm recovery of bioluminescent *S aureus* Xen36.

### Statistics

Data analysis was performed using the GraphPad Prism (V6) software package (GraphPad Software Inc, La Jolla, CA, USA). Briefly, in vivo bacterial burdens (measured as photons per second and calculated AUC values for each animal) were compared between controls and each treatment group using the Kruskal-Wallis test and Dunn’s post hoc analysis. Ex vivo bacterial burdens from tissue samples and implanted K-wires (measured as CFUs for each animal) were compared between controls and each treatment group using the Kruskal-Wallis test and Dunn’s post hoc analysis. Ex vivo bacterial burdens from tissue samples and implanted K-wires also were compared between same dose IV and IORA treatments using a two-tailed Mann Whitney test. The number of animals with culture-positive or negative K-wires was compared using Fisher’s exact test comparing same dose IV versus IORA treatment.

## Results

### Influence of Antibiotic Dosage and Route on Bacterial Burden (Biophotonic Imaging)

Biophotonic imaging showed lower levels of bioluminescent bacteria in all vancomycin-treated animals as early as 1 day after surgery (Table 1)(median bioluminescence, Control: 2.2 × 10^6^ [range, 7.2 × 10^5^–4.3 × 10^6^]; V110_IV_: 4.9 × 10^5^ [range, 2.5 × 10^5^–7.2 × 10^5^], difference of median: 1.7 × 10^6^, p = 0.0016; V25_IV_: 4.9 × 10^5^ [range, 3.6 × 10^5^–5.7 × 10^5^], difference of medians: 1.7 × 10^6^, p = 0.0028; V25_IORA_: 5.3 × 10^5^ [range, 4.73 × 10^5^–6.15 × 10^5^], difference of medians: 1.7 × 10^6^, p = 0.0148) (Fig. 3A). With the numbers available, there was no difference in bioluminescence between untreated animals and those treated with cefazolin (median bioluminescence, Control: 2.2 × 10^6^ [range, 7.15 × 10^5^–4.34 × 10^6^]; C100_IORA_: 6.2 × 10^5^ [range, 4.1 × 10^5^–1.1 × 10^6^], p = 0.0606; C100_IV_: 6.1 × 10^5^ [range, 3.68 × 10^5^–2.55 × 10^6^], p = 0.2335) (Fig. 3A).

**Table 1:** *Staphylococcus aureus* bioluminescence Day 1 after surgery for antibiotic treatment compared with no treatment

Treatment	Median (range)*	Difference of medians to control*	p value
Control	2.19 × 10^6^ (7.15 × 10^5^–4.34 × 10^6^)		
V110_IV_	4.90 × 10^5^ (2.51 × 10^5^–7.20 × 10^5^)	1.70 × 10^6^	0.0016
V25_IV_	4.94 × 10^5^ (3.60 × 10^5^–5.70 × 10^5^)	1.70 × 10^6^	0.0028
V25_IORA_	5.34 × 10^5^ (4.73 × 10^5^–6.15 × 10^5^)	1.66 × 10^6^	0.0148
C100_IORA_	6.21 × 10^5^ (4.14 × 10^5^–1.07 × 10^6^)	1.57 × 10^6^	0.0606
C100_IV_	6.06 × 10^5^ (3.68 × 10^5^–2.55 × 10^6^)	1.58 × 10^6^	0.2335

* Photons/second; C = cefazolin; V = vancomycin; IV = intravenous; IORA = intraosseous regional administration.

At 4 days after surgery, the bioluminescent signals from animals treated with a suboptimal concentration of vancomycin IV (V25_IV_) returned to near control levels. However, the bioluminescent signals obtained from animals administered high-dose systemic IV vancomycin (V110_IV_), low-dose regional intraosseous vancomycin (V25_IORA_), and regional intraosseous cefazolin (C100_IORA_), were lower than those from control animals at this time (median bioluminescence (Table 2): Control: 1.64 × 10^6^ [range, 7.76 × 10^5^–3.96 × 10^6^]; V110_IV_: 5.45 × 10^5^ [range, 4.30 × 10^5^–1.20 × 10^6^], difference of median: 1.10 × 10^6^, p = 0.013; V25_IV_: 1.13 × 10^6^ [range, 5.91 × 10^5^–1.40 × 10^6^], difference of median: 5.10 × 10^6^, p > 0.99; V25_IORA_: 5.14 × 10^5^ [range, 3.83 × 10^5^–8.96 × 10^5^], difference of medians: 1.13 × 10^6^, p = 0.0012; C100_IORA_: 6.18 × 10^5^ [range, 3.88 × 10^5^–1.17 × 10^6^], difference of medians: 1.02 × 10^6^, p = 0.0140; C100_IV_: 6.72 × 10^5^ [range, 4.63 × 10^5^–1.30 × 10^6^], difference of medians: 9.68 × 10^5^, p = 0.1015 (Fig. 3B). Likewise, AUC values calculated for the bioluminescence signals from treated mice throughout the experiment were approximately ¼ the value of those calculated for the untreated controls (median bioluminescence (Table 3): Control: 5.01 × 10^6^ [range, 3.30 × 10^6^–1.02 × 10^7^]; V110_IV_: 1.52 × 10^6^ [range, 9.93 × 10^5^–3.13 × 10^6^], difference of median: 3.49 × 10^6^, p = 0.0026; V25_IV_: 1.94 × 10^6^ [range, 1.75 × 10^6^–3.35 × 10^6^], difference of median: 3.07 × 10^6^, p = 0.4934; V25_IORA_: 1.51 × 10^6^ [range, 1.25 × 10^6^–2.43 × 10^6^], difference of medians: 3.50 × 10^6^, p = 0.0011; C100_IORA_: 1.55 × 10^6^ [range, 1.19 × 10^6^–2.35 × 10^6^], difference of medians: 3.46 × 10^6^, p = 0.0016; C100_IV_: 2.35 × 10^6^ [range, 1.44 × 10^6^–4.16 × 10^6^], difference of median: 2.66 × 10^6^, p = 0.2312) (Fig. 4).

**Table 2:** *Staphylococcus aureus* bioluminescence 4 days after surgery for antibiotic treatment compared with no treatment

Treatment	Median (range)*	Difference of medians to control*	p value
Control	1.64 × 10^6^ (7.76 × 10^5^–3.96 × 10^6^)		
V110_IV_	5.45 × 10^5^ (4.30 × 10^5^–1.20 × 10^6^)	1.10 × 10^6^	0.0126
V25_IV_	1.13 × 10^6^ (5.91 × 10^5^–1.40 × 10^6^)	5.10 × 10^5^	> 0.9999
V25_IORA_	5.14 × 10^5^ (3.83 × 10^5^–8.96 × 10^5^)	1.13 × 10^6^	0.0012
C100_IORA_	6.18 × 10^5^ (3.88 × 10^5^–1.17 × 10^6^)	1.02 × 10^6^	0.0140
C100_IV_	6.72 × 10^5^ (4.63 × 10^5^–1.30 × 10^6^)	9.68 × 10^5^	0.1015

* Photons/second; C = cefazolin; V = vancomycin; IV = intravenous; IORA = intraosseous regional administration.

**Table 3:** *Staphylococcus aureus* bioluminescence area under curve values during 4 days for antibiotic treatment compared with no treatment

Treatment	Median (range)	Difference of medians to control	p value
Control	5.01 × 10^6^ (3.30 × 10^6^–1.02 × 10^7^)		
V110_IV_	1.52 × 10^6^ (9.93 × 10^5^–3.13 × 10^6^)	3.49 × 10^6^	0.0026
V25_IV_	1.94 × 10^6^ (1.75 × 10^6^–3.35 × 10^6^)	3.07 × 10^6^	0.4934
V25_IORA_	1.51 × 10^6^ (1.25 × 10^6^–2.43 × 10^6^)	3.50 × 10^6^	0.0011
C100_IORA_	1.55 × 10^6^ (1.19 × 10^6^–2.35 × 10^6^)	3.46 × 10^6^	0.0016
C100_IV_	2.35 × 10^6^ (1.44 × 10^6^–4.16 × 10^6^)	2.66 × 10^6^	0.2312

V = vancomycin; C = cefazolin; IV = intravenous; IORA = intraosseous regional administration.

### Influence of Antibiotic Dosage and Route on *S aureus* Survival (CFU Counts)

**Fig. 5A–B:**
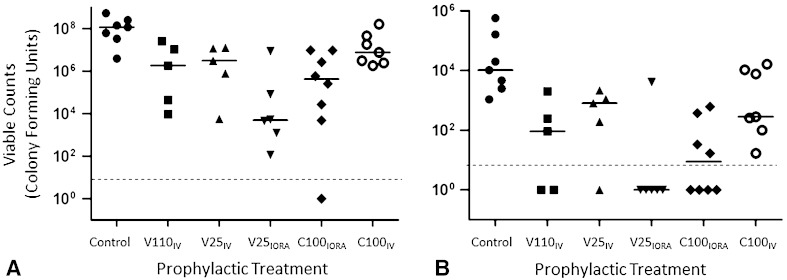
Quantification of viable *S aureus* Xen36 after surgery is shown. The mice were euthanized 96 hours after surgery for quantification of bacteria remaining in the (**A**) knee and surrounding tissue and (**B**) implanted K-wire. The dotted line represents the limits of detection. Median values per group are denoted by solid lines. Each symbol represents an individual animal. Data are pooled from six independent repeats with one to two animals per group per repeat. IV = intravenous; IORA = intraosseous regional administration; V110_IV_ = systemic vancomycin, 110 mg/kg; V25_IV_ = systemic vancomycin, 25 mg/kg; V25_IORA_ = IORA vancomycin, 25 mg/kg; C100_IORA_ = IORA cefazolin, 100 mg/kg; C100_IV_ = systemic cefazolin, 100 mg/kg.

Similar to data from biophotonic imaging, CFUs obtained from the implanted K-wire were lower in the high-dose systemic IV vancomycin, low-dose regional intraosseous vancomycin, and regional intraosseous cefazolin groups than controls (Table 4)(median CFUs, Control: 1.03 × 10^4^ [range, 1.08 × 10^3^–5.75 × 10^5^]; V110_IV_: 9.17 × 10^1^ [range, < 7.0 × 10^0^–2.00 × 10^3^], difference of median: 1.02 × 10^4^, p = 0.0313; V25_IV_: 4.96 × 10^2^ [range, < 7.0 × 10^0^–2.13 × 10^3^], difference of median: 9.80 × 10^3^, p = 0.0905; V25_IORA_: < 7.0 × 10^0^ [range, < 7.0 × 10^0^–4.08 × 10^3^], difference of medians: 1.03 × 10^4^, p = 0.0013; C100_IORA_: 8.85 × 10^0^ [range, < 7.0 × 10^0^–6.17 × 10^2^], difference of medians: 1.03 × 10^4^, p = 0.0020; C100_IV_: 2.83 × 10^2^ [range, 1.67 × 10^1^–1.62 × 10^4^], difference of median: 1.00 × 10^4^, p = 0.8858) (Fig. 5).

**Table 4:** *Staphylococcus aureus* recovered from implant 4 days after surgery* for effect of antibiotic treatment compared with no treatment

Treatment	Median (range)	Difference of medians to control	p value
Control	1.03 × 10^4^ (1.08 × 10^3^–5.75 × 10^5^)		
V110_IV_	9.17 × 10^1^ (< 7.0 × 10^0^–2.00 × 10^3^)	1.02 × 10^4^	0.0313
V25_IV_	4.96 × 10^2^ (< 7.0 × 10^0^–2.13 × 10^3^)	9.80 × 10^3^	0.0905
V25_IORA_	< 7.0 × 10^0^ (< 7.0 × 10^0^–4.08 × 10^3^)	1.03 × 10^4^	0.0013
C100_IORA_	8.85 × 10^0^ (< 7.0 × 10^0^–6.17 × 10^2^)	1.03 × 10^4^	0.0020
C100_IV_	2.83 × 10^2^ (1.67 × 10^1^–1.62 × 10^4^)	1.00 × 10^4^	0.8858

CFU = colony forming units; V = vancomycin; C = cefazolin; IV = intravenous; IORA = intraosseous regional administration.

Although bacteria were recovered from the tissues surrounding the implant site for all but one animal, mice treated with intraosseous vancomycin or cefazolin had lower numbers (Table 5): (median CFUs, Control: 1.17 × 10^8^ [range, 3.94 × 10^6^–5.37 × 10^8^]; V110_IV_: 1.86 × 10^6^ [range, 9.59 × 10^3^–2.60 × 10^7^], difference of median: 1.15 × 10^8^, p = 0.1376; V25_IV_: 1.95 × 10^6^ [range, 6.64 × 10^2^–1.27 × 10^7^], difference of median: 1.15 × 10^8^, p = 0.0454; V25_IORA_: 4.92 × 10^3^ [range, 1.16 × 10^2^–8.69 × 10^6^], difference of medians: 1.17 × 10^8^, p = 0.0005; C100_IORA_: 4.23 × 10^5^ [range, < 1.30 × 10^1^–9.69 × 10^6^] difference of medians: 1.17 × 10^8^, p = 0.0049; C100_IV_: 7.67 × 10^6^ [range, 1.82 × 10^6^–1.63 × 10^8^], difference of median: 1.09 × 10^8^, p = 0.8699 (Fig. 5).

**Table 5:** Staphylococcus aureus* recovered from periprosthetic tissue 4 days after surgery for effect of antibiotic treatment compared with no treatment

Treatment	Median (range)	Difference of medians to control	p value
Control	1.17 × 10^8^ (3.94 × 10^6^–5.37 × 10^8^)		
V110_IV_	1.86 × 10^6^ (9.59 × 10^3^–2.60 × 10^7^)	1.15 × 10^8^	0.1376
V25_IV_	1.95 × 10^6^ (6.64 × 10^2^–1.27 × 10^7^)	1.15 × 10^8^	0.0454
V25_IORA_	4.92 × 10^3^ (1.16 × 10^2^–8.69 × 10^6^)	1.17 × 10^8^	0.0005
C100_IORA_	4.23 × 10^5^ (< 1.30 × 10^1^–9.69 × 10^6^)	1.17 × 10^8^	0.0049
C100_IV_	7.67 × 10^6^ (1.82 × 10^6^–1.63 × 10^8^)	1.09 × 10^8^	0.8699

* Colony forming units; V = vancomycin; C = cefazolin; IV = intravenous; IORA – intraosseous regional administration.

### IORA versus Same-dose IV Antibiotic Administration

**Fig. 6A–B:**
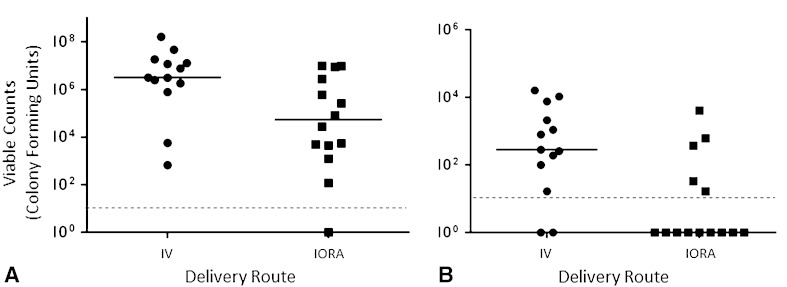
The effect of the delivery route of prophylactic treatment on *S aureus* Xen36 survival is shown. Mice treated with either 25 mg/kg vancomycin or 100 mg/kg cefazolin were euthanized 96 hours after surgery for quantification of bacteria remaining in the (**A**) knee and surrounding tissue and (**B**) implanted K-wire. The dotted line represents the limits of detection. Median values are denoted by solid lines. Each symbol represents an individual animal. Data are pooled from six independent repeats. IV = systemic administration; IORA = intraosseous regional administration.

Overall, intraosseous antibiotic administration was more effective at reducing the burden of contaminating bacteria in the tissue than the same dose of antibiotic administered IV (median CFUs, IV: 3.16 × 10^6^ [range, 6.64 × 10^2^–1.63 × 10^8^]; IORA: 5.43 × 10^4^ [range, < 1.30 × 10^1^–9.69 × 10^6^] difference of medians: 3.11 × 10^6^, p = 0.0163) (Fig. 6A). Bacteria were recovered from the K-wires implanted in only five of 14 IORA-treated animals compared with 11 of 13 animals treated intravenously with the same dose of antibiotic (Fisher’s exact p = 0.0183; median CFUs, IV: 2.83 × 10^2^ [range, < 7.0 × 10^0^–1.62 × 10^4^]; IORA: < 7.0 × 10^0^ [range, < 7.0 × 10^0^–4.08 × 10^3^] difference of medians: 2.76 × 10^2^, p = 0.0073) (Fig. 6B).

## Discussion

Prophylactic antibiotics reduce deep infection rates in arthroplasty [13, 23]. To be effective, prophylactic antibiotics must have adequate tissue concentrations at the operative site from the time of incision until the time of closure [5]. As antibiotic resistance increases, systemic administration of cephalosporins may no longer provide adequate tissue concentrations against coagulase-negative staphylococci and MRSA. IORA allows much higher tissue concentrations to be achieved [56, 57], and the current study showed that overall, IORA of cefazolin and vancomycin provided more effective prophylaxis than the same dose of antibiotic given systemically in a murine model of TKA.

There are a number of limitations to this study. First, although we attempted to use the equivalent antibiotic doses and copy the clinical situation of an intraarticular implant, it is unclear how well this model approximates the clinical situation of TKAs in humans. However because clinical TKA infection rates range between 0.86% and 2.5% [1, 3, 37, 41], animal models such as this remain the only practical way to provide adequate power to compare differing prophylaxis regimes. Second, we chose to investigate only MSSA, because vancomycin is likely to be more effective than cefazolin against coagulase-negative staphylococci and MRSA strains resistant to cefazolin. Similar to previous studies [38, 43], we used a relatively high inoculum of bacteria to better discriminate between the effectiveness of prophylactic regimes for the three endpoints used (in vivo bioluminescence, ex vivo implant, and periarticular tissue counts). This may differ from the clinical situation in TKA, because although contamination occurs in most if not all TKAs [12], the overall bacterial inoculum is likely to be lower than used in this model. In addition, vancomycin has a longer half-life than cefazolin which may have affected the comparison between groups as we used only one preoperative dose. However clinical data suggest the preoperative dose is the most important in providing prophylaxis [15, 16, 21, 51], and the faster rate of drug metabolism in the mouse means the effect of differing half-lives is reduced [44]. Finally we used only female mice, as male mice are more likely to fight and injure themselves or other animals when group housed. In murine models female mice generally are more resistant to the development of bacterial infection [40], however previous studies using this TKA model also have been single-sex studies [2, 38] and male and female differences are not known.

We found in vivo bacterial burden 4 days after simulated TKAs to be lower than that of controls in both IORA groups (low-dose vancomycin and standard-dose cefazolin), and also in the group given high-dose systemic vancomycin. The rationale for using a low-dose of vancomycin with IORA relates to the multiple disadvantages of systemic vancomycin prophylaxis. Systemic vancomycin requires a prolonged administration time to prevent red man syndrome, a pruritic, erythematous rash related to histamine release with rapid infusion [32, 48]. A prophylactic dose of 1 g requires the infusion to be started a minimum of 1 hour before surgery, which is difficult to achieve in an arthroplasty practice [4]. Vancomycin also can cause renal and other systemic toxicity [9, 32]. The use of a lower, targeted vancomycin dose through IORA optimizes timing of administration and reduces the risk of such systemic toxic effects.

Similar to data from biophotonic imaging, bacterial CFU counts from the implanted K-wire were lower than those of controls in both IORA groups, and in the group given high-dose systemic vancomycin. This suggests high tissue concentrations of vancomycin in particular are important in its efficacy as a prophylactic agent. The killing power of vancomycin is proportional to the area under the concentration versus time curve [45, 46]; thus, higher concentrations are likely to enhance efficacy, as seen in our study. Inadequate tissue concentrations have been implicated as the reason why systemic vancomycin is less effective than cephalosporins against MSSA [4, 42, 52]. Niska et al. [38] used a murine model of prophylaxis against implant infection to investigate the efficacy of varying doses of antibiotic. They found vancomycin to have a narrower effective dose range than daptomycin or tigecycline with a 110-mg/kg dose markedly more effective than a 10-mg/kg dose. Although estimation of equivalent human and mouse dosages is imperfect, our study supports the finding that the efficacy of vancomycin as a prophylactic agent depends on achieving high tissue concentrations. IORA vancomycin, which will achieve high concentrations despite the lower dose, resulted in lower bacterial counts and IORA vancomycin appeared at least as effective as cefazolin for prophylaxis against MSSA in our model. In clinical studies of prophylaxis in arthroplasty, vancomycin performs less well against MSSA than cephalosporins [4, 19]. It seems likely that the clinical efficacy of vancomycin prophylaxis against MSSA will be enhanced if higher tissue concentrations can be achieved.

We found the same doses of vancomycin and cefazolin were more effective via IORA than an IV dose. Bactericidal activity of cefazolin normally is considered to be concentration-independent, and once tissue levels are four to five times the minimum inhibitory concentration, further increases do not increase efficacy [10]. Therefore while high tissue concentrations of cefazolin with IORA may provide benefit against organisms with high minimum inhibitory concentrations of cefazolin such as coagulase-negative staphylococci [55], they would be expected to have less effect on more-sensitive strains such as the MSSA used in our study. However, these data are based on animal models of treatment of established infections [31, 53], rather than models of prophylaxis such as ours in which prevention of infection is the goal. Initiation of bacterial killing is known to occur earlier with increasing cefazolin concentrations [10], a factor likely to be more important in prophylaxis where preventing initial bacterial adherence and subsequent biofilm formation is required. This may explain our finding of greater efficacy for IORA cefazolin prophylaxis compared with systemic administration of the same cefazolin dose.

IORA of prophylactic cefazolin and vancomycin was more effective than the same dose of antibiotic given systemically. The effectiveness of vancomycin in particular was enhanced by IORA administration despite a lower IORA dose, suggesting vancomycin is more effective against MSSA when high tissue concentrations such as with IORA are achieved. Further clinical studies are needed to identify any unforeseen complications with IORA use, particularly with vancomycin. The use of a lower dose and depot effect may reduce the risk of red man syndrome on tourniquet deflation, and this complication has not yet been seen in human studies of IORA vancomycin. Concerns regarding antibiotic stewardship remain, and routine use of vancomycin by any route may not be justified. IORA vancomycin may be more appropriately limited to patients at higher risk of infection, such as with revision procedures, and in patients with a high BMI [35]. Future clinical studies will focus on these areas.
